# HPV16 Seropositivity and Subsequent HPV16 Infection Risk in a Naturally Infected Population: Comparison of Serological Assays

**DOI:** 10.1371/journal.pone.0053067

**Published:** 2013-01-02

**Authors:** Shih-Wen Lin, Arpita Ghosh, Carolina Porras, Sarah C. Markt, Ana Cecilia Rodriguez, Mark Schiffman, Sholom Wacholder, Troy J. Kemp, Ligia A. Pinto, Paula Gonzalez, Nicolas Wentzensen, Mark T. Esser, Katie Matys, Ariane Meuree, Wim Quint, Leen-Jan van Doorn, Rolando Herrero, Allan Hildesheim, Mahboobeh Safaeian, Costa Rican Vaccine Trial Group

**Affiliations:** 1 Division of Cancer Epidemiology and Genetics, National Cancer Institute, Bethesda, Maryland, United States of America; 2 Proyecto Epidemiológico Guanacaste, Fundación INCIENSA, Guanacaste, Costa Rica; 3 Department of Epidemiology, Harvard School of Public Health, Boston, Massachusetts, United States of America; 4 HPV Immunology Laboratory, SAIC-Frederick Inc., NCI-Frederick, Frederick, Maryland, United States of America; 5 MedImmune, Gaithersburg, Maryland, United States of America; 6 PPD Vaccines and Biologics Center of Excellence, Wayne, Pennsylvania, United States of America; 7 GlaxoSmithKline Biologicals, Rixensart, Belgium; 8 DDL Diagnostic Laboratory, Voorburg, The Netherlands; 9 Prevention and Implementation Group, International Agency for Research on Cancer, Lyon, France; National Institute of Health - National Cancer Institute, United States of America

## Abstract

**Background:**

Several serological assays have been developed to detect antibodies elicited against infections with oncogenic human papillomavirus (HPV) type 16. The association between antibody levels measured by various assays and subsequent HPV infection risk may differ. We compared HPV16-specific antibody levels previously measured by a virus-like particle (VLP)-based direct enzyme-linked immunoassay (ELISA) with levels measured by additional assays and evaluated the protection against HPV16 infection conferred at different levels of the assays.

**Methodology/Principal Findings:**

Replicate enrollment serum aliquots from 388 unvaccinated women in the control arm of the Costa Rica HPV vaccine trial were measured for HPV16 seropositivity using three serological assays: a VLP-based direct ELISA; a VLP-based competitive Luminex immunoassay (cLIA); and a secreted alkaline phosphatase protein neutralization assay (SEAP-NA). We assessed the association of assay seropositivity and risk of subsequent HPV16 infection over four years of follow-up by calculating sampling-adjusted odds ratios (OR) and HPV16 seropositivity based on standard cutoff from the cLIA was significantly associated with protection from subsequent HPV16 infection (OR = 0.48, CI = 0.27–0.86, compared with seronegatives). Compared with seronegatives, the highest seropositive tertile antibody levels from the direct ELISA (OR = 0.53, CI = 0.28–0.90) as well as the SEAP-NA (OR = 0.20, CI = 0.06, 0.64) were also significantly associated with protection from HPV16 infection.

**Conclusions/Significance:**

Enrollment HPV16 seropositivity by any of the three serological assays evaluated was associated with protection from subsequent infection, although cutoffs for immune protection were different. We defined the assays and seropositivity levels after natural infection that better measure and translate to protective immunity.

## Introduction

Infection with carcinogenic human papillomaviruses (HPV), most notably types 16 and 18, is necessary for the development of cervical cancer [1], the third most common cancer in women worldwide [2]. While infection with HPV is quite common, with the lifetime incidence estimated to be 80% [3], most infections become undetectable within 1–2 years [4]. Only a small fraction of infections with high-risk HPV fail to clear, resulting in overt HPV persistence [5]. Persistent HPV infection is strongly associated with the development of cervical intraepithelial neoplasia grade 3 (CIN3), which progresses to invasive cervical cancer in a minority of cases [6]. Immune responses generated upon HPV infection are likely to be a critical mechanism for preventing, controlling, and eliminating HPV infection [7].

Characterization of the immune responses to HPV virions can help explain how prior infection or immunization protects against future infection and associated disease. Correlates of protection are currently still unclear. Neutralizing antibodies are expected to be the primary immune mechanism for protection against HPV infection [8]. Because of the role of antibodies in preventing HPV infections, serological assays are important for measuring the antibodies or other immune factors directed against HPV, and these assays may identify the individuals who had mounted an immune response to previous exposure to HPV and may be protected against subsequent HPV infection [9].

Serological assays for HPV16 based on different biochemistry include the competitive Luminex immunoassay (cLIA), designed to measure antibodies against a specific neutralizing epitope [10]; the secreted alkaline phosphatase neutralization assay, designed to measure overall neutralizing potential, (SEAP-NA) [11]; and the virus-like particle (VLP)-based enzyme-linked immunosorbent assay (ELISA), designed to measure a broad spectrum of neutralizing and non-neutralizing antibodies directed against the L1 capsid protein [12]. Each assay provides only a partial characterization of immune status, and comparison of seroprevalence across assays is complicated because the assays differ quantitatively, i.e., by throughput and detection range, and qualitatively, i.e., whether they detect antibodies against multiple epitopes, which may be indicative of previous exposure, or neutralizing antibodies that may confer immune protection. Immune responses to HPV measured by currently available assays may or may not predict individual protection [13], but limited work has been done to determine if any measured immune response can define immediate or future protection against HPV infection or associated disease [9], [14], [15].

Safaeian et al. showed that women who have the highest seropositive tertile of HPV16 and HPV18 antibody levels based on a VLP-based direct ELISA assay are significantly protected from subsequent infection with HPV16 and HPV18, respectively [14], after controlling for risk factors associated with newly detected HPV infection. Due to the potential for serological assays to differentially measure HPV antibody levels in association with protection from subsequent HPV infection, we compared the previous HPV16 results obtained using a VLP-based direct ELISA with results obtained using a VLP-based cLIA and a SEAP-NA. Here, we aimed to identify the assays and seropositivity levels after natural infection that better measure and translate to protective immunity.

## Materials and Methods

### Study Population

We selected serum samples obtained at enrollment visit prior to vaccination from women in the control arm of the Costa Rica HPV16/18 Vaccine Trial (CVT), a publicly funded, randomized trial of the efficacy of the HPV16/18 vaccine manufactured by GlaxoSmithKline (GSK) for the prevention of HPV16/18 infection and related precancerous lesions among women. Informed consent was received from subjects in the trial (registered as Clinical Trial number NCT00128661), and the details of the trial and characteristics of the trial participants have been described elsewhere [16], [17]. All study protocols were reviewed and approved by the National Cancer Institute (NCI) and Costa Rican Institutional Review Boards.

### Biospecimens

Prior to randomization and vaccination, pelvic examinations were conducted on all sexually experienced women, and cervical cells were collected and placed in liquid medium (PreservCyt; Cytyc Corporation, now Hologic, Marlborough, MA) for liquid-based cytology (Thin-Prep; Cytyc Corporation, now Hologic) and for cervical HPV detection. Blood was collected on all participants, and serum samples were used for HPV serological testing by direct ELISA as part of the trial. After vaccination, women were followed-up annually. At each follow-up visit, cervical samples were collected from sexually experienced women for HPV DNA testing. The SPF10-HPV LiPA25 version 1 system (Labo Biomedical Product, Rijswijk, The Netherlands) was used for all HPV DNA testing, as previously described [14].

### Stratified Sampling Strategy

**Figure 1:**
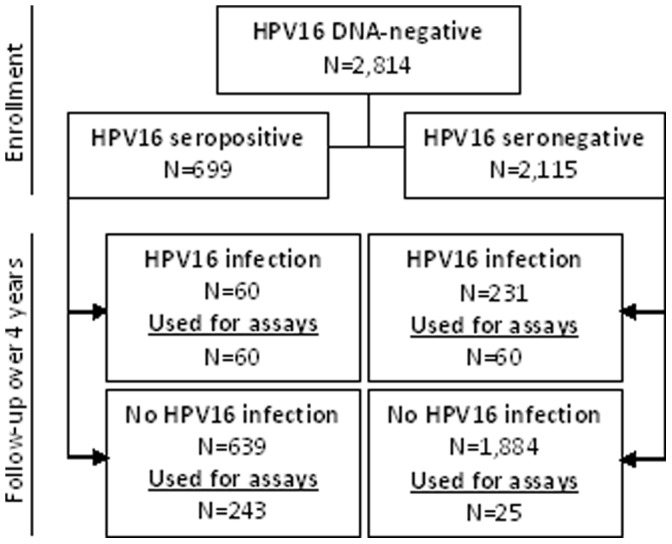
Consort diagram of the study population and targeted sampling strategy for HPV16 serological assays study. Out of the 639 women, who were HPV16 seropositive by ELISA at enrollment and did not develop HPV16 infection over follow-up in the population, 35 did not have enough sample volume for testing and were excluded when drawing samples.

The economical sampling strategy (Figure 1) selected 388 women from 2,814 HPV16 DNA-negative women. Sampling strata were defined based on HPV16 serology as measured by ELISA and incident HPV16 infection. Among 2,814 HPV16 DNA-negative women at enrollment in the control arm who had follow-up HPV test results, 699 women were categorized as HPV16 seropositive and 2,115 as seronegative by the VLP-based direct ELISA at enrollment. Among the HPV16 seropositive women, we included all 60 individuals who developed an incident HPV16 infection and 60 women who were HPV16 seronegative at enrollment and developed an incident HPV16 infection. We included a subset of individuals who did not develop an incident HPV16 infection from among the women who were HPV16 seropositive (n = 243) and among those who were HPV16 seronegative (n = 25) at enrollment. This targeted sampling captured a wide range of ELISA titers, enabling us to compare the HPV16 direct ELISA against the cLIA and SEAP-NA [18] and to explore which assay best predicts protection from subsequent incident HPV16 infection during four years of follow-up. Identical enrollment prevaccination serum samples from these 388 participants were tested by all three assays.

### Serology Assays

All women included in this study had previous HPV16 serological measurements based on direct ELISA assays [14], a standard measure of immunogenicity through polyclonal antibodies, performed at GSK Biological in Rixensart, Belgium, as described previously [19], [20]. The standard cutoff for seropositivity by the HPV16 VLP-based direct ELISA serology results was 8 EU/mL. The cLIA assay was performed as previously described at PPD Vaccines, Biologics and Biomarkers (Wayne, PA), and the standard seropositivity cutoff used for HPV16 was 20 mMU/mL [21]. The SEAP-NA was performed as previously described at the NCI-SAIC HPV Immunology Laboratory (Frederick, MD) [11], and the standard seropositivity cutoff used for the HPV16 SEAP-NA was 25.1.

### Statistical Analysis

The results from each assay were dichotomized as seropositive or seronegative based on laboratory-based standard cutoffs as defined above. In addition, the seropositive samples were further categorized as “low,” “medium,” and “high” based on tertiles. Because our sampling scheme was based on the prior knowledge of the ELISA results and new infection status, to avoid potentially biased estimates, we derived all of our population-based estimates by using inverse-probability weights to account for the stratified sampling scheme. The sampling fraction for a stratum is the ratio of numbers of stratum members in the stratified sample and in the cohort. The reciprocal of the sampling fraction for a stratum served as the weight for every individual in the stratum. Mann-Whitney p-values were calculated for the comparison of sampling-adjusted medians. To study the association between assay seropositivity and subsequent risk of HPV infection, we derived sampling-adjusted odds ratios (ORs) and 95% CIs from weighted logistic regression. All statistical analyses were run using SAS (v 9.2) and R (v 2.11.1).

## Results

**Figure 2:**
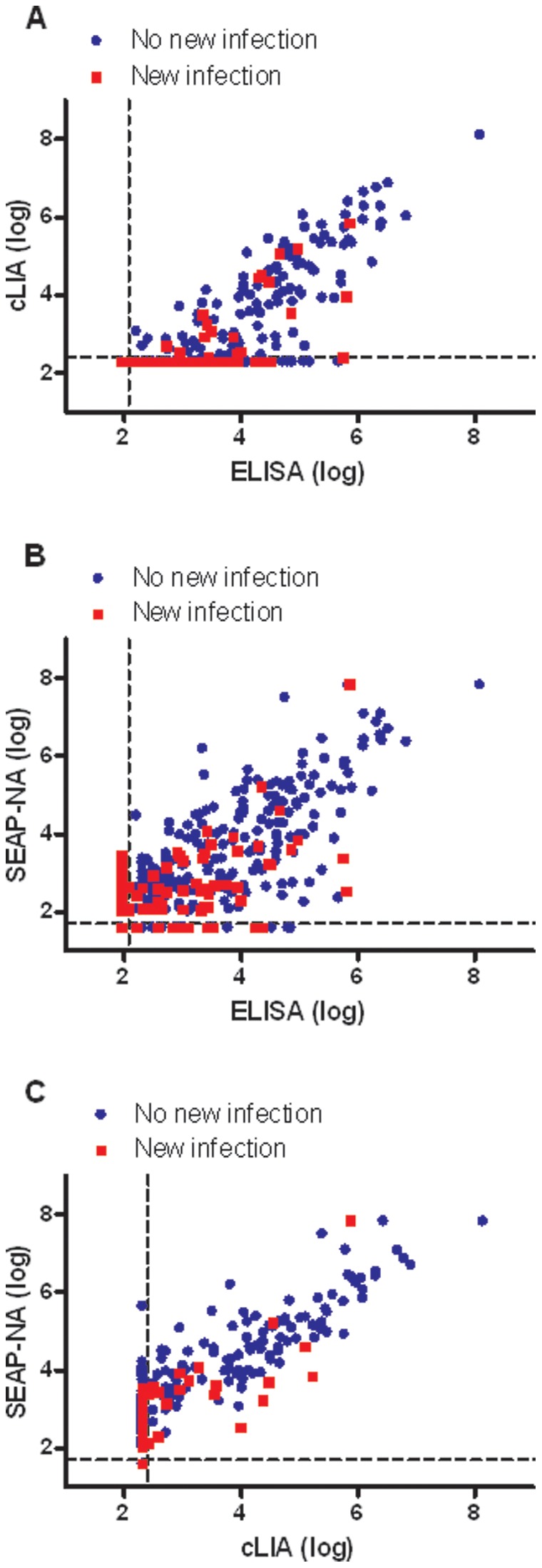
Graphical presentation of the results comparing each assay by new infection status. The results are plotted on the log scale for each assay; the dashed lines represent the standard cutoffs for seropositivity for the assays. (A, B, C) Blue points represent enrollment serology results for those subjects who did not have an incident HPV16 infection, and red squares represent enrollment serology results for subjects for whom we detected an incident HPV16 infection. New infection was detected over four years of follow-up.

Table 1 briefly summarizes the HPV16 serology results for the 388 subjects by incident HPV16 DNA infection status. The subjects’ minimum assay values did not differ by incident HPV16 infection status. However, the maximum assay values as well as the sampling-adjusted median values for subjects who did not develop a new incident infection were at least as high (SEAP-NA) or higher (ELISA and cLIA) than the maximum assay values for subjects who did develop a new incident infection. Those who did not develop a new incident infection had similar or higher sampling-adjusted median assay values compared with those subjects who did develop a new incident infection (Mann-Whitney p-values: ELISA, 0.15; cLIA, 0.29; SEAP-NA, <0.05). Graphical presentation of the results comparing each assay by new infection status is shown in Figure 2.

**Table 1:** HPV16 serology results by incident HPV16 DNA infection status for the 388 subjects selected for assay measurement.

	All subjects (N = 388)				No new infection (N = 268)				New infection (N = 120)			
Assay	Min	Max	Geometric mean^a^	Median^a^	Min	Max	Geometric mean^a^	Median^a^	Min	Max	Geometric mean^a^	Median^a^
ELISA	<8	3202	6.79	<8	<8	3202	6.90	<8	<8	345	5.97	<8
cLIA	<11	3370	6.91	<11	<11	3370	7.00	<11	<11	351	6.21	<11
SEAP-NA	5	2560	11.97	12.31	5	2560	12.22	13.12	5	2560	10.05	9.03

ELISA: VLP-based direct enzyme-linked immunosorbent assay; cLIA: Competitive Luminex immunoassay; SEAP-NA: Secreted alkaline phosphatase protein neutralization assay.

Values less than the level of quantification are shown as “<level of quantification”.

a Sampling-adjusted population estimates for the original cohort estimated using Inverse-probability weights (see methods). For calculation of sampling-adjusted values, the assay values under the level of quantification were assumed to be midway in the range. For example, ELISA values <8 were assumed to be 4, and cLIA values <11 were assumed to be 5.5.

We then examined the association between enrollment HPV16 serology based on laboratory-based standard seropositivity cutoffs and risk of incident HPV16 infection during four years of follow-up (Table 2). Using these sampling-adjusted population estimates, we found that 9% of the individuals who were categorized as seropositive using laboratory-based standard cutoffs by the direct ELISA, 5% by the cLIA, and 7% by the SEAP-NA developed an incident infection; by contrast, 11% of individuals who were categorized as seronegative by each assay developed an incident infection. Enrollment HPV16 seropositivity by cLIA was significantly associated with protection from incident HPV16 infection (OR = 0.48, 95% CI = 0.27–0.86, compared with seronegatives). However, as we found in our previous report [14], seropositivity using the standard cutoff for the direct ELISA was not associated with risk of incident HPV16 infection (OR = 0.77, 95% CI = 0.57–1.03, compared with seronegatives). Seropositivity using the standard cutoff for the SEAP-NA was also not associated with risk of incident HPV16 infection (OR = 0.66, 95% CI = 0.31–1.41, compared with seronegatives). In an analysis to consider the potential for cross-protection, we found that seropositivity using standard cutoffs for each of the three assays was not associated with risk of incident HPV31 infection (data not shown). We also examined these associations stratified by enrollment age and enrollment lifetime number of sexual partners and did not observe any differences by these strata (data not shown).

**Table 2:** Comparison of HPV16 serology assays at enrollment and protection against incident HPV16 DNA infections during four years of follow-up.

	Enrollment Serology^a^	Incident HPV16 infection		
Assay		New infection, N (%^b^)	No new infection, N (%^b^)	OR (95% CI)
ELISA	Negative	60 (11)	25 (89)	0.77 (0.57–1.03)^c^
	Positive	60 (9)	243 (91)	
cLIA	Negative	109 (11)	183 (89)	0.48 (0.27–0.86)^b^
	Positive	11 (5)	85 (95)	
SEAP-NA	Negative	100 (11)	151 (89)	0.66 (0.31–1.41)^b^
	Positive	20 (7)	117 (93)	

ELISA: VLP-based direct enzyme-linked immunosorbent assay; cLIA: Competitive Luminex immunoassay; SEAP-NA: Secreted alkaline phosphatase protein neutralization assay.

a Enrollment serology based on laboratory-based standard seropositivity cutoffs: ELISA, 8 EU/mL; cLIA, 20 mMU/mL; SEAP-NA, 25.1.

b Sampling-adjusted population estimates (see methods).

c Based on data from [14].

We further analyzed the enrollment HPV16 serological assay results that were considered seropositive in tertiles and their association with risk of incident HPV16 infection during four years of follow-up (Table 3). In our previous report [14], we found that compared with seronegative subjects, those who had the highest seropositive tertile by ELISA were significantly protected from incident infection. Here, we found that those subjects who had the highest seropositive tertile by SEAP-NA were also significantly protected from incident HPV16 infection. While seropositivity by the standard cutoff for the cLIA was associated with protection from incident infection, further stratification of positive samples into tertiles did not improve the risk prediction for the cLIA assay.

**Table 3:** Odds ratios and 95% confidence intervals for seropositive tertile categorizations of HPV16 assay results at baseline and risk of incident HPV16 DNA infection.

	Enrollment Serology^a^	Incident HPV16 infection		
Assay		New infection, N (%^b^)	No new infection, N (%^b^)	OR (95% CI)
ELISA	Negative	60 (11)	25 (89)	ref
	Low	20 (9)	73 (91)	0.81 (0.50–1.25)^c^
	Medium	28 (12)	86 (88)	0.95 (0.63–1.38)^c^
	High	12 (5)	84 (95)	0.53 (0.28–0.90)^c^
cLIA	Negative	109 (11)	183 (89)	ref
	Low	4 (6)	25 (94)	0.51 (0.18–1.43)^b^
	Medium	4 (6)	28 (94)	0.54 (0.19–1.50)^b^
	High	3 (5)	32 (95)	0.40 (0.12–1.26)^b^
SEAP-NA	Negative	100 (11)	151 (89)	ref
	Low	8 (13)	14 (87)	1.22 (0.24–6.31)^b^
	Medium	9 (7)	46 (93)	0.62 (0.31–1.23)^b^
	High	3 (2)	57 (98)	0.20 (0.06–0.64)^b^

ELISA: VLP-based direct enzyme-linked immunosorbent assay; cLIA: Competitive Luminex immunoassay; SEAP-NA: Secreted alkaline phosphatase protein neutralization assay.

a Categorization cutoffs for the ELISA: Low 8–16, Medium >16–59, High >59; CLIA: Low 20–52, Medium >52–120, High >120; SEAP-NA assay: Low 25–32.63, Medium >32.63–93.07, High >93.07.

b Sampling-adjusted population estimates (see methods).

c Previously reported in [14].

Lastly, we examined the association between enrollment HPV16 seropositivity by multiple assays and the risk of incident HPV16 infection (Table S1A). Compared with the subjects who were categorized as seronegative by all three assays, those subjects who were seropositive by all three assays (using standard cutoffs) had a significantly lower risk of developing an incident infection.

In our previous work [18], we determined that an alternate seropositivity cutoff of 60 EU/mL for the direct ELISA leads to better concordance between the ELISA and cLIA (at standard cutoff). Those who were seropositive using this alternate seropositivity cutoff for the ELISA had a lower risk of developing an incident infection (OR = 0.57, 95% CI = 0.33–0.99). Moreover, we also examined the strata of enrollment serology using this alternate seropositivity cutoff for the ELISA (Table S1B) and found similar results as before, such that subjects who were seropositive by all three assays had a significantly lower risk of developing an incident infection (OR = 0.42, 95% CI = 0.20–0.89).

## Discussion

Here, we evaluate the association between HPV16-specific immune responses generated following natural infection by three different serological assays and risk of subsequent HPV16 infection. We observed a significant association between enrollment HPV16 seropositivity and protection from subsequent incident HPV16 infection over four years of follow-up; however, the extent of protection was dependent on data categorization for individual serological assays. Women whose levels were above the standard cutoff for the cLIA had half the risk of acquiring an incident infection of seronegative women. The risk of subsequent infection in women with enrollment ELISA or SEAP-NA results in the highest seropositive tertile was significantly lower than that of seronegative women. Also, women who were seropositive by all three assays were less likely to acquire a subsequent incident infection than women who were seronegative by all assays.

The currently available assays we used in this study are heterogeneous. Our point estimates of protection for cLIA and SEAP-NA assays were both stronger than that for the ELISA assay. As a polyclonal antibody assay, the VLP-based direct ELISA measures total serum anti-VLP IgG antibodies to all epitopes presented by VLPs, and therefore, the ELISA was designed to measure total antibodies and may be best for measuring previous exposure to HPV, as explored in another study of this population [22]. The ELISA does not differentiate between type-specific conformational antibodies and type-common antibodies, which tend to be specific for epitopes presented by denatured L1 capsid protein and are usually not neutralizing [23].

By contrast, the cLIA was designed to measure epitope-specific antibodies; as a monoclonal antibody assay, the cLIA was developed to specifically detect antibodies that target one immunodominant epitope of HPV16 L1 (the V5 epitope). In our study, the significant association between cLIA seropositivity based on standard cutoffs and protection is an indication that the cLIA is an assay that captures a more specific measure of protective immunity compared with the ELISA and SEAP-NA. These results suggest that the cLIA, using laboratory-determined, standard seropositivity cutoffs, measures a subset of the overall polyclonal responses measured by the ELISA. Such findings are similar to those of a previous study that compared the ELISA and cLIA and found the assays to be well correlated [15]; however, this paper is the first to show correlation between the ELISA and cLIA among non-immunized individuals.

The cLIA may not measure neutralizing antibodies as comprehensively as the SEAP-NA because the cLIA is a limited assay that measures a single, albeit immunodominant, neutralizing epitope. However, in our study, the standard seropositivity cutoff for the SEAP-NA was not significantly associated with protection from incident infection, which is surprising considering that it is a biological assay that measures neutralizing potential regardless of immune component or mechanism and is considered the gold standard for measuring protection through the in vivo neutralization potential of serum. However, as a complex biological assay, the SEAP-NA suffers from the limitation of being less reproducible (i.e., higher % CVs) than the ELISA and Luminex-based assays.

A challenge in finding an appropriate HPV serological assay to determine a threshold or correlate of protective immunity is that some naturally infected individuals who do not become seropositive using any currently available assay could still be protected against disease following later re-infection, possibly due to cellular immune responses [10]. Moreover, repeated exposure to HPV may lead to stronger responses, but results from a serologic assay do not provide a history of cumulative exposure. In our study, subjects may have been previously exposed to HPV16 but developed protective immune responses that were not measured by any of the serological assays; such misclassification would have biased our results to the null. In addition, we cannot determine whether women who were HPV16 DNA-negative were actually exposed, although the numbers of recent sexual partners were the same in the infected and non-infected groups [14]. Nonetheless, we can only measure the “failures” (those for whom we detected a new infection) and derive indirect measures of protection. Another limitation is that we do not know if the new infections over follow-up were the result of actual new infections or reactivated infections.

Biologic complexities hinder interpreting current HPV serology findings. Protective immunity may be associated not only with antibody levels but also with antibody affinity and avidity, which are not measured by the assays used in this study. The correlation between cell-mediated immunity and antibody levels may explain some of the observed associations between antibody assays and protective immunity to subsequent infection; at the same time, the weakness of the correlation may explain the low ability of antibody levels to identify who is protected from subsequent infection.

We used data and serum collected from the control arm of a community-based vaccine trial that included women who were selected from a population census. Rather than measuring the serum samples from all the eligible HPV16-DNA-negative women in the control arm by all three serological assays (n ∼8400 assays), we designed this study using knowledge of prior results that indicated that participants with elevated HPV16 antibody levels (by ELISA) following natural infection had a significant 50% reduced risk of subsequent new HPV16 infection compared with seronegatives. Therefore, we selected women based on their enrollment ELISA seropositivity and their newly detected HPV16 infections over the four-year follow-up period. While the limitation was that all samples were selected based on seropositivity by the ELISA, this sampling strategy was efficient, cost-effective, and allowed for inferences to be applied to the original control arm. To account for the sampling strategy and avoid biased estimates, we applied an adjustment to all estimates that were weighted to represent the entire cohort. Antibody levels do not fully explain subsequent protection or susceptibility for HPV infection, so there is a continued need for valid and reliable assays to better determine correlates of protection. Future research efforts should be directed toward correlating measures of virus type-specific immunity with protection against re-infection with these types and toward determining the duration of potential cross-protection from natural immunity.

## Supporting Information

Table S1 **Risk of HPV16 DNA infection in strata of enrollment HPV16 ELISA, cLIA, and SEAP-NA serology**
(DOC)
