# Supplementary material for: HPV16 Seropositivity and Subsequent HPV16 Infection Risk in a Naturally Infected Population: Comparison of Serological Assays
Source: PLoS One. 2013 Jan 2;8(1):e53067. doi: 10.1371/journal.pone.0053067 (PMC3534652; doi:10.1371/journal.pone.0053067)
Supplement: Table S1 — Risk of HPV16 DNA infection in strata of enrollment HPV16 ELISA, cLIA, and SEAP-NA serology (DOC) [file pone.0053067.s001.doc]

| **Table S1. Risk of HPV16 DNA infection in strata of enrollment HPV16 ELISA, cLIA, and SEAP-NA serology**  **A** | | | | | |
| --- | --- | --- | --- | --- | --- |
| ELISA (standard cutoff^a^) | CLIA (standard cutoff^a^) | SEAP-NA (standard cutoff^a^) | N | Incident infection, N (%^b^) | OR (95% CI)^b^ |
| Negative | Negative | Negative | 81 | 57 (11) | ref |
|  | Negative | Positive | 4 | 3 (13) | 1.26 (0.12-13.29) |
|  |  |  |  |  |  |
| Positive | Negative | Negative | 168 | 42 (11) | 0.97 (0.78,-1.21) |
|  | Positive | Negative | 2 | 1 (34) | 4.24 (0.26-67.95) |
|  | Negative | Positive | 39 | 7 (7) | 0.64 (0.29-1.39) |
|  | Positive | Positive | 94 | 10 (5) | 0.44 (0.24-0.79) |
| **B** |  |  |  |  |  |
| ELISA (alternate cutoff^a^) | CLIA (standard cutoff^a^) | SEAP-NA (standard cutoff^a^) | N | Incident infection, N (%^b^) | OR (95% CI)^b^ |
| Negative | Negative | Negative | 234 | 96 (11) | ref |
|  | Negative | Positive | 37 | 9 (10) | 0.94 (0.25-3.59) |
|  | Positive | Positive | 21 | 3 (5) | 0.47 (0.14-1.59) |
|  |  |  |  |  |  |
| Positive | Negative | Negative | 15 | 3 (10) | 0.95 (0.27-3.33) |
|  | Positive | Negative | 2 | 1 (34) | 4.26 (0.27-68.49) |
|  | Negative | Positive | 6 | 1 (8) | 0.71 (0.08-6.16) |
|  | Positive | Positive | 73 | 7 (5) | 0.42 (0.20-0.89) |
| ELISA: VLP-based direct enzyme-linked immunosorbent assay; cLIA: Competitive Luminex immunoassay; SEAP-NA: Secreted alkaline phosphatase protein neutralization assay | | | | | |
| ^a^Enrollment serology based on laboratory-based standard seropositivity cutoffs: ELISA, 8 EU/mL; cLIA, 20 mMU/mL; SEAP-NA, 25.1; alternative seropositivity cutoff: ELISA, 60 EU/mL | | | | | |
| ^b^Sampling-adjusted population estimates (see methods) | | | | | |
